# Insights from China: understanding the impact of community resilience and government trust in psychological resilience and anxiety during COVID-19

**DOI:** 10.3389/fpubh.2023.1298269

**Published:** 2023-11-28

**Authors:** Yue Hu, Yuxin Huang, Hua Zhang, Min Fang, Guobang Chen

**Affiliations:** ^1^School of Political Science and Public Administration, Guangxi Minzu University, Guangxi, China; ^2^School of Public Administration, South China Agricultural University, Guangzhou, Guangdong Province, China

**Keywords:** COVID-19, anxiety, psychological resilience, community resilience, government trust, mental well-being

## Abstract

**Background:**

COVID-19 has the potential to greatly impact an individual mental well-being. However, an individual’s psychological resilience, combined with support from their community and government disaster relief efforts can aid individuals in confronting crises with a positive mindset. The purpose of this study is to investigate how individuals, across three dimensions of individual resilience perception, community resilience perception, and government trust perception, mitigate individual anxiety during COVID-19.

**Methods:**

This study employed an online survey method that was not restricted by geographical location. Data collection took place from January 2022 to June 2022, and the valid questionnaires covered all 31 provinces, autonomous regions, and municipalities in China. The assessment of community resilience was conducted employing the Conjoint Community Resilience Assessment Measure-10 (CCRAM-10). Structural Equation Modeling (SEM) was also used to examine the relationship between community resilience, government trust, individual psychological resilience, and anxiety.

**Results:**

The SEM results reveal that individual psychological resilience is significantly negatively correlated with anxiety (*b* = −0.099, *p* < 0.001), while there is a significant positive correlation between community resilience perception (*b* = 0.403, *p* < 0.001) and government trust (*b* = 0.364, *p* < 0.001) with individual psychological resilience. Furthermore, government trust perception enhances psychological resilience, consequently reducing anxiety (*b* = −0.036, *p* < 0.001). The results also revealed that women and increasing age had a mitigating effect on individual anxiety during COVID-19.

**Conclusion:**

Individual’s mental state is influenced on multiple dimensions during COVID-19. Not only can individual psychological resilience better cope with anxiety, but support at the community and government dimensions has a significant impact on individual psychology. These resources can enhance the resilience of both individuals and communities, helping them better cope with stress and difficulties.

## Introduction

Ulrich Beck’s risk society thesis underscores that contemporary society is characterized by an awareness of risk and uncertainty (1). During a pandemic outbreak, individuals may experience heightened levels of anxiety, fear, helplessness, and stress related to the possibility of getting sick or dying (1–3). No individual can easily avoid exposure to public risks. Moreover, with many countries implementing stay-at-home measures to reduce the spread of the novel coronavirus, social interactions among residents have decreased. During this time, individuals may experience increased social isolation and loneliness, leading to more pronounced levels of anxiety and depression (4–7). Research has unveiled that psychological resilience plays a pivotal role in enabling individuals to adapt to their psychological, emotional, and physical environments while facilitating self-recovery and rejuvenation following periods of duress. This intrinsic psychological resilience is instrumental in an individual’s capacity to confront diverse stressors and challenges, thereby augmenting self-assurance, optimism, and overall life quality (8–10).

However, during a crisis, vulnerable individuals have limited capacities to cope with risks and have limited access to human an economic resource that can be mobilized (11). In such circumstances, the importance of external support in reducing an individual’s vulnerability to further trauma becomes paramount (12). Primarily, communities first become the buffer point under the impact of public crises. Social support from neighbors and friends within residential communities significantly reduces the negative impact of major disasters on individual mental health (13). In addition, citizens’ confidence in local government can diminish the public perception of crisis-related risks and future apprehensions, thus augmenting their perceived control over the crisis and effectively safeguarding their mental health (11, 14).

Research related to resilience have attracted the attention of numerous disciplines (15). However, current research on community resilience primarily concentrates on the resilience capacity at the community level (9, 16–18). Secondly, previous studies have often examined pairwise relationships, such as the impact of community resilience on psychological resilience or the relationship between trust in government and mental health. Using SEM, a commonly used tool in psychological research. It better allows for the examination and identification of the correlations and the proportion of mediating effects among variables. This study employs SEM to examine how assessments in three dimensions, namely individual resilience, perceived community resilience, and perceived government trust, affect mental health during COVID-19.

### Anxiety and psychology resilience during crisis

Anxiety is a common human psychological emotion, typically triggered by both internal and external stimuli. In appropriate circumstances, anxiety responses can help individuals better cope with stress and challenges. However, excessive anxiety can lead to various psychological disturbances, subsequently affecting an individual’s physiological and behavioral well-being (19). During a pandemic outbreak, individuals may experience fear and a sense of helplessness regarding illness or death (20, 21). Feelings of social isolation and loneliness may intensify, and the levels of anxiety and depression may become more pronounced (5–7).

Psychological resilience explains why certain individuals are better able to process traumatic internal injuries than others (22), achieving better psychological and emotional balance (23, 24), and being more likely to respond positively during crises (25). Psychological resilience can be seen as a malleable capability, which is a person’s capacity to adapt and recover when facing difficulties, setbacks, and stress. This capability can change with changes in the environment (26). This ability can be learned and developed by anyone (27). Many studies have confirmed that this inherent psychological resilience is crucial for an individual’s ability to confront diverse stresses and challenges, contributing to increased self-confidence, motivation, and quality of life (8, 10, 28).

Prior literature has demonstrated a negative relationship between psychological resilience and anxiety. For instance, studies on events such as Hurricane Katrina in Louisiana and the Deepwater Horizon oil spill have shown that lower levels of psychological resilience in individuals are associated with higher rates of depression and anxiety (29). Another example is the aftermath of intensive terrorist attacks in Israel, where individual resilience serves as a protective factor, effectively reducing individual anxiety levels (17).

### Community resilience during crisis

The development of individual psychological resilience is not only associated with individual characteristics but also closely related to one’s social support network. A positive and supportive peer group can provide necessary support and assistance, thereby enhancing an individual’s psychological resilience (12). While individual resilience plays a role in coping with crises, individuals in crisis situations, especially vulnerable groups, are often more susceptible to risk, making external support crucial in minimizing the risk of further trauma. During public crises, communities become a buffer in the face of crisis impacts, serving as the frontline units in dealing with the crisis directly, responding to it, and managing it (30).

Community resilience is an ability that encompasses both resilience and protection (31). Researchers have pointed out that resilience plays a role in maintaining stability, recovering, and reconstructing (32). These abilities and functions stem from the community itself and are reflected in its members (33). A resilient community not only helps prevent or minimize loss or damage to life, property, and the environment but also has the capacity to respond quickly and recover normal operations, even when critical parts of the community are severely affected (34). Communities can increase their resilience, reduce risk, and overall vulnerability through sustainable development policies, effective intervention measures, increased social support and resources (35, 36).

Communities are the refuge for residents, especially resilient communities that can effectively reduce the impact of disasters on residents, provide timely assistance and support, and help people gradually return to normal life. Resilient communities can provide emotional support, material assistance, and social connections, offering strong support for individual resilience during crises (37). When communities successfully resist risks, people’s psychological stress is relieved (38), thus reducing the trauma caused by risks and communication errors (39). Social support from neighbors and friends in residential communities also significantly reduces the negative impact of major natural disasters on individuals’ mental health (13). Braun-Lewensohn and Sagy (40) found that community resilience is closely related to the reduction of anxiety levels among rural residents during missile attacks. Williams and Merten’s research (41) discovered that community interactions among teenagers have a positive impact on the long-term mental health of teenagers.

### Government crisis management and trust

Government’s governance actions during disasters and citizens’ trust in the government can also have a positive impact on the development of psychological resilience (14). Behavioral public administration applies psychological theories to introduce government trust as a factor influencing individual psychology in public crises (42, 43). The logic behind how government trust alleviates individual anxiety during crises is as follows: firstly, citizens’ trust in the government can reduce the public’s perception of crisis-related risks and future concerns, enhance their perception of crisis controllability, thus effectively protecting their mental health (14, 44, 45). Studies have pointed out that local governments played a crucial role in issuing policies, communicating information, and organizing resources during the Covid-19 crisis (30). During the outbreak of the SARS virus in 2003, the trust of residents in the Hong Kong region in the government and healthcare institutions effectively mitigated the harm caused by personal anxiety (14). Under the influence of government and media protective measures, residents’ trust reduces their perceived risk, weakening the sense of crisis (46). When the public has trust in the government, they are more likely to accept the information and measures provided by the government, thus reducing unnecessary panic and worry (44).

Moreover, as a mechanism for reducing complexity, public trust in the government can also increase cooperation and coordination between the government and the public, maintaining people’s ability to act in complex environments, thereby better addressing crises (47). Because crisis events provide opportunities that require close social cooperation to address them, positive outcomes in crisis interventions can lead to a “unity effect” in public psychology (48). Therefore, trust is a key element in resolving collective action dilemmas (49). Government trust also increases community cooperation, thus enhancing community resilience.

Secondly, the policies issued by the government are mainly implemented at the community dimension, with communities in China designed as the basic administrative units. Community resilience plays a supportive role in individual resilience, and the construction of community resilience also requires support and efforts from various stakeholders. This includes support and assistance from local governments, non-governmental organizations, and other relevant stakeholders (50). Community resilience requires sufficiently strong and fast resources to facilitate functional recovery in response to changing environments (15). Some researchers have pointed out that resilient communities are successful in lobbying the government to provide resources for community reconstruction (51). The higher the level of material preparedness, the higher the perception of residents regarding the connections, resources, and potential for change within the community (52). When resources and characteristics are sufficient to generate resistance or resilience, the community can maintain its functionality (36).

### Framework and hypotheses

**Figure 1:**
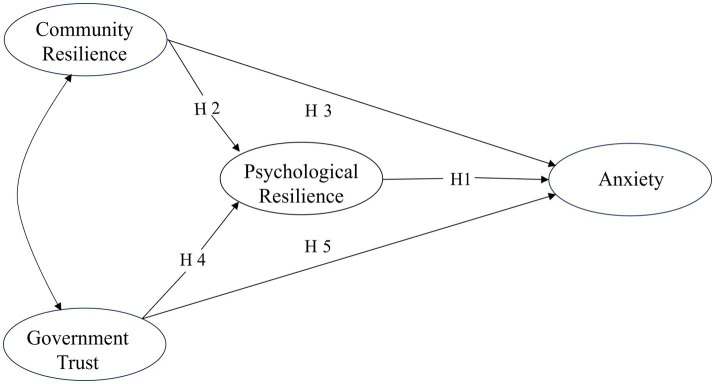
The theoretical framework that encompasses the relationships between interconnects anxiety, psychological resilience, community resilience, and government trust.

The above literature emphasizes the impact of community resilience and government trust on individual psychological resilience and anxiety. Based on the literature, we have established a theoretical framework for anxiety, psychological resilience, community resilience, and government trust (Figure 1). First, researchers have pointed out that psychological resilience explains why some individuals are better able to cope with traumatic injuries than others (22), making it easier to achieve psychological and emotional balance (23, 24, 53). Therefore, we propose the hypothesis:

*Hypothesis 1 (H1)*: Individual psychological resilience mitigates individual anxiety emotions.

Furthermore, research indicates that community resilience has a positive impact on individual psychological resilience. The stress-buffering hypothesis confirms that social support may positively affect individuals’ psychological ability to resist risks during crises, thereby moderating the impact of stress on pathological stress responses (54). The more support from external sources, the stronger an individual’s ability to cope with stressful situations (37).

In addition to the influence of community resilience on psychological resilience, research also suggests the role of community resilience in psychological well-being. Personal social support enhances an individual’s ability to cope with external challenges, and the individual’s sense of anxiety during crises is reduced (55). Social support from neighbors and friends in residential communities also significantly reduces the negative impact of major natural disasters on individual mental health (13). Zhang et al.’s (56) study found that international students in Wuhan, during the lockdown, experienced reduced anxiety through the indirect impact of perceiving community resilience on community communication and support. Zhang et al.’s (18) research found that community resilience alleviated mental health stress among the older adults through the perception of community prevention effects. Based on the literature mentioned above, we propose the following hypotheses:

*Hypothesis 2 (H2)*: Community resilience increases individual psychological resilience.

*Hypothesis 3 (H3)*: Community resilience alleviates anxiety by enhancing individual psychological resilience.

Individual trust in the government can have a positive impact on the development of psychological resilience (11). When the public has trust in the government, it enhances their perception of crisis controllability and can further effectively protect their mental health (14, 46). Previous research has often not strictly distinguished psychological well-being from psychological resilience and anxiety. We believe that trust in the government not only enhances individual resilience and reduces individual anxiety but also alleviates anxiety through the enhancement of individual resilience. Based on this, we propose the following hypotheses:

*Hypothesis 4 (H4)*: Citizens’ government trust enhances individual psychological resilience.

*Hypothesis 5 (H5)*: Government trust alleviates anxiety by enhancing individual psychological resilience.

Furthermore, community resilience builds a stable institutional environment that encourages the formation of trust beliefs and trust behaviors. Similarly, trust is conducive to the occurrence of cooperative behavior (57), and when such cooperative behavior occurs within a community, community resilience is also enhanced. Trust and community resilience mutually influence each other, leading to co-variation effects and impacting individual resilience. Zhang’s (58) study treated trust in the government as a moderating variable for community resilience and anxiety. Community resilience reduced anxiety in older adults during COVID-19, but this association weakened in older adults with low trust in the government. In another study by Zhang, governance efficacy was treated as an intermediate variable for community resilience (18). We believe that communities and higher-level local governments are different government levels that residents can typically distinguish and perceive their subjective feelings. Therefore, we consider the perceived community resilience at the community dimension and the perceived trust in the government at the government dimension as two independent variables. The subjective feelings of these two government dimensions will have co-variation effects.

## Methods

### Variables and measurement

The first section of the questionnaire comprises demographic information about residents, including gender, age, marital status, household registration, political affiliation, educational level, and annual income. The second section assesses anxiety, psychological resilience, community resilience, and government trust.

#### Anxiety

Drawing from the model of the Generalized Anxiety Disorder-7 (GAD-7) scale (59), we measured anxiety using a 7-item anxiety subscale from the Depression Anxiety Stress Scales. Participants were asked to indicate how much time, in the past 2 weeks, they have been troubled by specific issues presented in 7 questions. The seven questions are: “1. Feeling tense, anxious, or restless.” “2. Unable to stop or control worries.” “3. Excessive worry about various things.” “4. Finding it difficult to relax.” “5. Unable to calm down due to unease.” “6. Easily getting upset or irritable.” “7. Feeling something dreadful is going to happen.” Responses were scored on a scale from 1 to 4, with higher scores indicating greater levels of anxiety.

#### Psychological resilience

Psychological resilience refers to an individual’s ability to adapt to external environmental changes when faced with adversity, threats, or challenges through self-regulation or external support (12). This study assessed participants’ self-perceived level of psychological resilience under emergency circumstances using two questions: “1. I can adapt to change” and “2. I tend to recover quickly after illness or difficulties.” Responses were rated on a scale from 1 to 5.

#### Community resilience

Community resilience capacity is defined as a social system’s preparedness, response, and recovery capabilities when faced with destructive disaster events (31). Assessment indicators for community resilience encompass a community’s ability to resist, respond to, recover from, and rebuild after crisis events (36). We adopted the CCRAM-10 assessment framework, which serves as a comprehensive indicator reflecting a community’s crisis response and recovery capabilities. It assesses community resilience from five aspects. Leadership: “1. The local government of my community functions well” and “2. The decision makers in the local government handle matters appropriately.” Collective efficacy: “3. There are mutual assistance and people care for one another. “and “4. I count on my community to assist and share essential information.” Preparedness: “5. Community has well-established infrastructure for emergency situation “and 6. Residents are aware of their roles in an emergency and respond promptly “. Place attachment: “7. I am proud to tell others where I live and participate community issues “and “8. I have a sense of belonging to my community.” Social trust: “9. Good relationships exist between various groups “and “10. Residents in my community trust each other and community develop well “. This framework is a well-established tool for assessing urban community resilience (60).

Specifically, leadership covers the cognitive perception of positive support provided by community leaders from the top down. Collective efficacy represents the level of mutual assistance and concern among neighbors. Preparedness involves the awareness of the community’s ability to respond to emergencies. Local attachment represents residents’ identification with their own community. Social trust reflects mutual trust and relationships among community residents (60). In this study, a 5-point Likert scale was used to measure the 10 items, with higher scores indicating a stronger perceived sense of community resilience.

#### Government trust

Government trust refers to the trust and reliance that the public places in the government. This trust is based on the belief in the government’s ability, goodwill, and integrity (61). To assess residents’ trust in the government during emergencies, we employed a 5-point Likert scale and asked:"1. Are you satisfied with the central government? “and “2. Are your satisfaction with the local government?” These items assessed the degree of trust residents had in government, with higher scores indicating greater trust.

### Sample and data collection

This study used a questionnaire survey method, and data collection took place from January 2022 to June 2022, collecting a total of 2,316 questionnaires. During the COVID-19 pandemic, due to the widespread implementation of social distancing measures, conducting in-person surveys became challenging. Therefore, this study employed an online survey method that was not limited by geographical location. Participants were contacted using a snowball sampling method through the internet and social media, and data collection was conducted through anonymous online questionnaires. To select participants, we used the general characteristics of the entire online population as a reference. We chose four main demographic variables, including gender, region, educational level, and household registration, as sampling criteria. Researchers on social media selected respondents who met these criteria and distributed online questionnaires to them to obtain the sample. Valid questionnaires covered all 31 provinces, autonomous regions, and municipalities in China. Since we conducted an online convenience survey, the participants were relatively younger, but their characteristics were similar to those of Chinese internet users. After excluding invalid questionnaires, we obtained 2,279 valid questionnaires.

### Data analysis

We commenced by conducting a descriptive statistical analysis of socio-demographic characteristics among our 2,279 participants, covering variables such as gender, age, marital status, residence, political affiliation, education, and income. Secondly, we conducted a correlation analysis to investigate potential associations between socio-demographic factors and our measurement variables. Finally, we employed SEM to examine the mediating role of psychological resilience in the relationships between anxiety and both community resilience and government trust. This analytical process encompassed model development, parameter estimation, and model fit testing, all executed using STATA 15.1 software.

## Results

### Descriptive statistics

The study included 2,279 participants, with a higher proportion of female participants (*n* = 1,334, 58.53%) compared to male participants (*n* = 945, 41.47%). The mean age was 28.66 years, with a median age of 24 years. Regarding marital status, 65.60% were unmarried, while 34.40% were married. In the context of political alignment, 21.59% were identified as members of the Chinese Communist Party (hereinafter referred to as CCP Members), while the vast majority, constituting 78.41%, were non-members of the Chinese Communist Party (hereinafter referred to as Non-CCP Members). Household registration was categorized as urban (*n* = 1,200, 52.65%) and non-urban (*n* = 1,079, 47.35%). Education levels were divided into three categories: high school and below (*n* = 297, 13.03%), college and bachelor’s degree (*n* = 1,697, 74.46%), and postgraduate or higher (*n* = 285, 12.51%; see Table 1).

**Table 1:** Demographic characteristics of the participants.

Variables	The meaning and assignment of variables	Mean	SD	N	Percent (%)	Min/Max
Gender	Female (0)			1,334	58.53	
	Male (1)			945	41.47	
Age		28.66	10.64	2,279	100	18/72
Marital Status	Singl (0)			1,495	65.6	
	Married (1)			784	34.4	
Household registration	Non-urban (0)			1,079	47.35	
	Urban (1)			1,200	52.65	
Political status	Non-CCP Member (0)			1787	78.41	
	CCP Member (1)			492	21.59	
Education Level	Below high school			297	13.03	
	Associate and bachelor’s degree			1,697	74.46	
	Postgraduate			285	12.51	
Annual income	Below 50,000Yuan			1,398	61.34	
	50,001–100,000 Yuan			487	21.37	
	100,001–200,000 Yuan			269	11.8	
	20,001–500,000 Yuan			94	4.12	
	More 500,001 Yuan			31	1.36	

The measurement of the four variables, anxiety, psychological resilience, community resilience, and government trust are carried out using 5-Likert scales. Firstly, Cronbach’s alpha was employed to examine the reliability of anxiety (α = 0.968), psychological resilience (α = 0.840), community resilience (α = 0.973), and government trust (α = 0.836). The Cronbach’s alpha values for the core variables were all greater than 0.80, which validates the high internal consistency of the relevant items on this scale, indicating good reliability. When we do correlation analysis and SEM later, we normalize the variables of 1–5 or 1–4 (Table 2).

**Table 2:** Investigation items of core variables in questionnaire.

Variables	Items of questionnaire survey	Mean(SD) normalization	α
Anxiety (GAD-7)	1. Feeling tense, anxious, or restless.	0.511(0.239)	0.968
	2. Unable to stop or control worries.	0.466(0.239)	
	3. Excessive worry about various things.	0.488(0.235)	
	4. Finding it difficult to relax.	0.467(0.239)	
	5. Unable to calm down due to unease.	0.429(0.239)	
	6. Easily getting upset or irritable.	0.460(0.235)	
	7. Feeling something dreadful is going to happen.	0.426(0.237)	
Psychological resilience	1. I can adapt to change.	0.865(0.153)	0.840
	2. After difficulties, I tend to recover quickly.	0.830(0.172)	
Community resilience (CCRAM-10)	1. The local government of my community functions well.	0.834(0.195)	0.973
	2. The decision makers in the local government handle matters appropriately.	0.846(0.184)	
	3. There is mutual assistance and people care for one another.	0.836(0.191)	
	4. I count on my community to assist and share essential information.	0.857(0.180)	
	5.Community has well-established infrastructure for emergency situation.	0.845(0.183)	
	6. Residents are aware of their roles in an emergency and respond promptly.	0.836(0.187)	
	7. I am proud to tell others where I live and participate community issues.	0.821(0.203)	
	8. I have a sense of belonging to my community.	0.838(0.178)	
	9. Good relationships exist between various groups.	0.829(0.191)	
	10.Residents in my community trust each other and community develop well.	0.842(0.183)	
Government trust	1. Are you satisfied with the central government?	0.918(0.137)	0.836
	2. Are your satisfaction with the local government?	0.881(0.165)	

### Analysis of SEM results

In this study, we employed SEM for analysis. The model’s fit indices are as follows: the chi-square value is 4038.35 with 323 degrees of freedom, the Comparative Fit Index (CFI) is 0.934, the Tucker-Lewis Index (TLI) is 0.928, the Standardized Root Mean Square Residual (SRMR) is 0.041, and the Root Mean Square Error of Approximation (RMSEA) is 0.071. All these indicators meet the relevant standards and requirements, indicating a good model fit.

**Figure 2:**
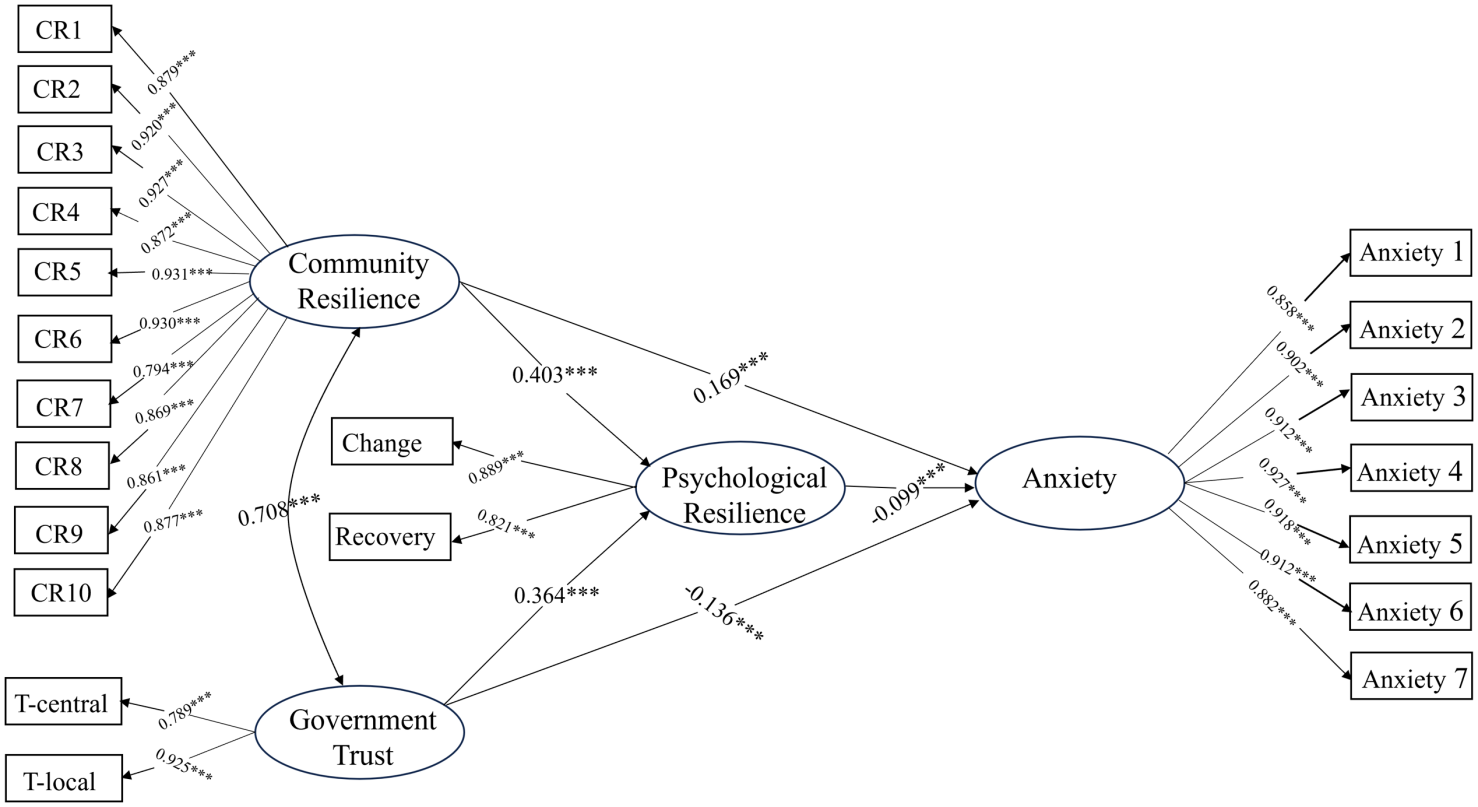
The results of SEM.

The results (see Figure 2; Table 3) show a significant negative correlation between anxiety and psychological resilience (*b* = −0.099, *p* < 0.001), confirming hypothesis H1. The enhancement of psychological resilience mitigates the negative impact of anxiety.

**Table 3:** The results of direct, indirect, and total effects of demographic variables, anxiety, psychological resilience, community resilience, and government trust.

		Direct effects (SE)	Indirect effects (SE)	Total effects (SE)
Psychological	Community resilience	0.403***(0.022)		0.403***(0.022)
resilience	Government trust	0.364***(0.037)		0.364***(0.037)
Anxiety	Psychological Resilience	−0.099***(0.053)		−0.099***(0.053)
	Community Resilience	0.169***(0.042)	−0.040***(0.018)	0.129***(0.039)
	Government Trust	−0.136***(0.070)	−0.036***(0.025)	−0.172***(0.065)
	Male	0.063***(0.009)		0.063***(0.009)
	Age	−0.110***(0.001)		−0.110***(0.001)
	Married	−0.018(0.014)		−0.018(0.014)
	City	0.022(0.009)		0.022(0.009)
	CCP Member	0.021(0.012)		0.021(0.012)
	Educational Level	−0.036(0.014)		−0.036(0.014)
	Annual Income	0.013(0.006)		0.013(0.006)

*p < 0.05; **p < 0.01; ***p < 0.001.

There is a significant positive correlation between community resilience and psychological resilience (*b* = 0.403, *p* < 0.001), confirming hypothesis H2. This suggests that a stronger community resilience is associated with higher individual psychological resilience in the face of risks. The construction of community resilience has a positive effect on individuals in risk situations. Community resilience has a positive and significant effect on anxiety (*b* = 0.169, *p* < 0.001). Although community resilience reduces anxiety by increasing psychological resilience (*b* = −0.040, *p* < 0.001), the overall effect remains significantly positive (*b* = 0.129, *p* < 0.001), and hypothesis H3 is not supported.

Individual government trust is significantly positively correlated with psychological resilience (*b* = 0.364, *p* < 0.001), confirming hypothesis H4. Residents with high trust in the government exhibit greater psychological resilience. Government trust not only reduces anxiety (*b* = −0.136, *p* < 0.001) but also alleviates anxiety by enhancing individual psychological resilience (*b* = −0.036, *p* < 0.001). The total effect of government trust on anxiety is −0.172 (*p* < 0.001), confirming hypothesis H5.

The results of the covariate relationship between community resilience and government trust indicate a significant association between the two. There is a positive relationship between community resilience and government trust, with a standardized coefficient of *b* = 0.71 (*p* < 0.001), highlighting the significant positive correlation between increased community resilience and higher levels of government trust. This underscores the important connection between community resilience and government trust.

Regarding demographic variables, males were found to be more anxious than females (*b* = 0.063, *p* < 0.001). Increasing age (*b* = −0.110, *p* < 0.001) significantly mitigated anxiety and had a positive impact on psychological well-being. Educational level (*b* = −0.036, *p* = 0.147), household registration (*b* = 0.022, *p* = 0.339), marital status (*b* = −0.018, *p* = 0.581), and income (*b* = 0.013, *p* = 0.604) had no significant impact on anxiety.

## Discussion

During times of crises, individuals consistently endeavor to establish supportive connections with others. When self-reliant individuals become part of a collective entity, it substantially contributes to the accomplishment of objectives previously unattainable on an individual basis (13). Integration into social networks can aid an individual in avoiding adverse experiences, thereby augmenting the likelihood of psychological resilience (55), subsequently bolstering one’s psychological resilience. This assimilation into social networks progressively nurtures a communal sense of efficacy, laying the groundwork for an individual’s psychological flexibility and recovery (12). Social support encompasses emotional, informational, material, and cognitive facets, among others. These resources have the potential to fortify the resilience of both individuals and communities, enabling them to better cope with and adapt to stress and adversity (62). Our research demonstrates that individual resilience during COVID-19 is influenced by community resilience and government trust, thereby affecting individual psychological well-being.

### Individual dimension – psychological resilience can alleviate anxiety

Psychological resilience acts as a safeguarding element for residents and augmenting psychological resilience can mitigate the adverse effects of anxiety. In times of peril, “fear and apprehension” are common manifestations among individuals navigating a state of existential risk. Concerned about their “ontological security,” individuals may grapple with “existential anxiety,” prompting them to either vacate the hazardous zone or mitigate harm (63). Psychological resilience is crucial for an individual’s ability to cope with various stressors and challenges, promoting overall adaptation and mental well-being (8, 10). This implies that individuals in adversity can overcome difficulties through their own efforts. Our findings also reinforce previous research conclusions that high psychological resilience fosters the development of positive cognitions about oneself, the world, and the future, reducing anxiety during COVID-19.

### Community resilience – its impact on individual resilience and anxiety

The pressure-buffering hypothesis suggests that social support has a positive impact on individuals’ ability to resist risk during crises (54). Crises hold a dual significance for communities. When managed effectively, crises can activate advantages and exhibit a diminishing effect; conversely, mismanagement can lead to amplification within a disaster chain reaction (64). Communities, serving as buffers during crises, play two crucial roles in crisis periods. Firstly, they constitute the fundamental unit of governance, and their performance in emergency functions directly extends and supplements the government’s crisis management capabilities, underpinning the overall crisis management of society (65, 66). Researchers have also pointed out the role of communities in soft mobilization during crises. Public crisis management represents an extraordinary mode of governance, where the administrative and political mobilization methods effective in routine management may become less efficient. Effective self-mobilization within society can transmit government decisions and crisis-related knowledge to grassroots and individuals, aiding in dispelling panic induced by crises and enhancing societal and individual crisis resilience (67).

The construction of community resilience encompasses the accumulation of diverse social capital, which provides support to individuals during risks. The more social support individuals receive during risks, the stronger their psychological resilience against risks becomes. Our data results reveal that community resilience also acts as a ‘protective umbrella’ for individuals. Resilient communities develop their own resources in social, political, cultural, and psychological aspects to mitigate the impact of risks on the community (68). They may even utilize crises in reverse, strengthening their pre-existing resilience and perpetuating a self-enhancing environment for the community (31). Given that communities are on the frontline of risk, the construction of their resilience is particularly essential in supporting individuals. Individuals facing risks can seek external assistance through community social networks to acquire risk information and leverage community resources to enhance their adaptability to risks, thus reducing panic and anxiety arising from a lack of control or understanding of external circumstances.

Our results indicate a positive and significant relationship between community resilience and anxiety, with community resilience not mitigating anxiety through individual resilience. Previous research has often confirmed the positive relationship between community resilience and individual mental health, such as Zhang’s study (18), which found that community resilience alleviated mental health stress among the older adults by perceiving community preventive effects. Nevertheless, most studies have not directly demonstrated the relationship between community resilience and anxiety. Another study by Zhang (56) found that international students in Wuhan during the lockdown period were indirectly influenced by community resilience perception through community communication and support to alleviate anxiety, but community resilience perception did not have a direct effect on anxiety. Lee et al. (9) found that community resilience could enhance individual psychological resilience, but the relationship between community resilience and anxiety was not significant. Lyons et al. (51), through correlation analysis, identified a positive relationship between community resilience and higher psychological well-being but did not control for other variables using multiple regression. Williams and Merten’s research (41) revealed that increased community interactions among adolescents fostered their agency and significantly reduced depressive symptoms, but the direct impact of community interactions on anxiety symptoms was positively significant. Our results confirm the substantial effect of communities on individual resilience but do not alleviate anxiety. Given the multifactorial nature of anxiety, variables such as trust in the government and individual resilience play a significant role in mitigating anxiety. The SEM analysis clearly demonstrates the contributions of variables to anxiety relief, both indirectly and directly.

### Government trust – its impact on individual resilience and anxiety

The conclusion reveals that government trust not only significantly positively influences individual psychological resilience but also plays a constructive role in alleviating anxiety. When individuals encounter difficulties, seeking assistance and collaborating with others can help them better cope with challenges and enhance their survival and recovery capabilities. In situations with a high level of external pressure controllability, individuals facing risks become more resilient in terms of risk tolerance and recovery capabilities. Trust is a key factor in individuals’ actions during risk, and higher levels of trust lead individuals to actively seek external support to gain greater pressure controllability. This sense of unity can be achieved through the establishment of trust and common goals, thereby assisting individuals and groups in coping with stress and challenges (12, 69).

Mutual trust and dependence between the government and residents are among the political characteristics of emergency management in China (70). Firstly, in China, disaster management power is largely concentrated in the hands of the central government, which plays a crucial role in disaster reduction, preparedness, and response. Secondly, under the influence of cultural factors related to legitimacy, residents’ trust in the government significantly influences their risk perception. This trust and confidence are primarily affected by the government’s ability and performance in crisis prevention and management. When the government is well-prepared, efficient, and responsive, citizens do not excessively worry about crises, and they are less critical of related crisis management decisions (30). Furthermore, local governments can provide necessary resources and support to enhance community disaster preparedness, response, and resilience. Community resilience built on the foundation of robust community resources is beneficial for community resistance to external crises and can serve as a “safe haven” for individuals during public crises.

In times of crisis impact, mutual trust, and a sense of solidarity among people play a crucial and positive role in subsequent disaster management (38). Good crisis governance by the government enhances citizens’ trust in the government during crises (47). When the public has trust in the government, they are more likely to accept the information and measures provided by the government, thereby reducing unnecessary panic and worry. Additionally, public trust in the government can also increase cooperation and coordination between the government and the public, enabling a better response to crises. These factors contribute to improving individual psychological resilience and alleviating anxiety. Previous research has pointed out the “unity effect,” (48) which is attributed to the belief that being part of a group can provide individuals with many benefits. As a member of a group, an individual can access social support and resources, thereby increasing their chances of survival and psychological recovery. Our research confirms this. Specifically, government trust is a protective factor for individuals. Enhanced trust in the government strengthens the impact of individual psychological resilience on mental health.

### The mutual influence between community resilience and government trust

The construction of community resilience also requires support and efforts from various stakeholders, with many resources relying on local and central government provision for community rebuilding (15, 36, 52). Our findings underscore the close relationship between community resilience and government trust. Highly trusted communities often form bonds of mutual assistance, which can provide residents with robust emotional support and reduce their fear of risks (71). A resilient community, by definition, implies strong social support, a sense of trust, and robust stability and rebuilding capabilities. Residents coexisting in a public crisis within such a community can utilize the abundant social capital and social networks within the community to regulate their own anxiety in the face of unexpected situations, thereby enhancing their individual psychological resilience.

## Limitations

The limitations of this study are as follows. Firstly, due to the convenience sampling method used in the study, the representativeness of the questionnaire survey participants was affected, limiting the generalizability of the conclusions. Secondly, the heterogeneity of communities has a significant impact on individuals residing within them, and community resilience is related to the type and characteristics of the community, which can clearly influence individuals living in the community. Whether this influence has structural characteristics is a variable that was not addressed in this study and therefore cannot be analyzed. Inequality in residence and its impact on individuals is a topic worthy of future attention.

## Conclusion

In this study, the factors influencing individual anxiety in a major crisis were examined, and the research distinguished how evaluations at the individual, community, and government dimension interacted and affected mental health. The results indicate that in a super crisis, individual psychology is impacted on multiple dimensions. Not only does individual psychological resilience better cope with anxiety in the crisis, but support at the community and government dimensions also significantly affects individual psychology. For more vulnerable individuals in times of risk, enhanced trust, and sense of belonging among community members facilitate the effectiveness and quality of social support, thereby strengthening self-regulation and self-recovery capabilities at both the community and individual dimensions. Additionally, trust during risk contributes to the formation of cooperative behaviors, allowing individuals to mitigate the impact of risk and subsequently alleviate anxiety, enhancing psychological resilience. Our study reinforces this conclusion. Particularly in the context of China, government governance actions and public trust in the government are strong influencing factors on individuals’ psychology. Trust in the government during risk enhances individual psychological resilience, thus mitigating anxiety. This expands our understanding of the impact of community and government governance as external environmental factors on mental health in the context of major crises. Thirdly, the study employed SEM, which helped us to delineate the interrelationships among subjective variables and their contributions to the dependent variable.

## Data availability statement

The raw data supporting the conclusions of this article will be made available by the authors, without undue reservation.

## Ethics statement

The studies involving humans were approved by Academic Ethics Committee of School of Politics Science and Public Administration, Guangxi Minzu University. The studies were conducted in accordance with the local legislation and institutional requirements. The participants provided their written informed consent to participate in this study.

## Author contributions

YHu: Data curation, Investigation, Methodology, Software, Writing – original draft, Writing – review & editing. YHua: Data curation, Methodology, Writing – original draft, Investigation, Software. HZ: Data curation, Methodology, Writing – original draft, Conceptualization, Funding acquisition, Supervision, Writing – review & editing. MF: Conceptualization, Data curation, Methodology, Writing – review & editing. GC: Data curation, Software, Writing – review & editing.
